# Correlations between family characteristics and childcare in optimizing the growth of children under six years

**DOI:** 10.1186/s12889-025-21931-0

**Published:** 2025-02-27

**Authors:** Laili Rahayuwati, Desy Indra Yani, Sri Hendrawati, Arlette Suzy Setiawan, Damar Irza, Sherllina Rizqi Fauziah

**Affiliations:** 1 Department of Community Nursing, Faculty of Nursing, Universitas Padjadjaran, Sumedang, West Java Indonesia; 2 Department of Pediatric Nursing, Faculty of Nursing, Universitas Padjadjaran, Sumedang, West Java Indonesia; 3 Department of Pediatric Dentistry, Faculty of Dentistry, Universitas Padjadjaran, Sumedang, West Java Indonesia; 4 Nursing Science Study Program, Faculty of Nursing, Universitas Padjadjaran, Sumedang, West Java Indonesia

**Keywords:** Childcare, Children, Development, Family, Growth

## Abstract

**Background:**

Families have a the primary influence on children. In particular, excellent childcare in the family is assumed to correlate with children’s health status, growth, and development. Hence, some family factors contribute to the optimization of childcare. This study aims to analyze family characteristics that correlate with childcare for children under six in Indonesia.

**Methods:**

The data collection instrument used was a Performance and Accountability Survey Program (SKAP) questionnaire of the National Population and Family Planning Board for Indonesia to assess family function in children’s health development in a sample of 7,651 parents, mothers, and/or fathers of children under six years of age. Childcare was the outcome variable. In addition, the chi-square test and logistic regression were used for statistical analysis.

**Results:**

The results showed a significant correlation between parents’ education, family health insurance ownership, number of toddlers, preschool age children and media exposure to health with family parenting patterns that influence children’s health. Meanwhile, parental age and parents occupation did not significantly contribute to childcare quality. Multivariate analysis showed that older age (particularly 20–34 years), exposure to media about health, family without health insurance, and parents with more than two toddlers and pre-school age were associated with better childcare.

**Conclusions:**

Several family characteristics were significantly associated with childcare for children under six, including health media exposure, older parental age (particularly 20–34 years), do not have family health insurance and have more than two toddlers and pre-school age children. These things contribute to the fulfilment of nutrition and exclusive breastfeeding, parenting patterns, and providing access to health services for children in the family.

**Ethics application number:**

The data were approved by the National Population and Family Planning Board for Indonesia Ethical Review Institutions number 454/LB.02/H4/2019.

## Background

Family is an element that can directly correlate with the status of individual health, especially that of children [1]. The family is a provider of economic, social, and psychological resources and serves to protect it from health threats. Family is the primary influence on children, included in many preschool children. According to Duvall’s 1962 theory, there are eight stages in the family life cycle, with the third stage being families with preschool children, where the oldest child is between 2 ½ and 6 years old [2]. Family factors, such as resources and parenting patterns that shaped healthy lifestyles in the past, influenced lifestyles later in life [3]. In addition, environmental and family factors can influence the behavior of parents and children. Specifically, family characteristics, including access to socioeconomic resources, such as education level and income, are associated with differences in parenting styles for childcare. As such, parents with educational backgrounds below high school, lack of support, and lack of resources are more likely to have authoritarian and permissive parenting styles [4]. Higher income, scholarly performance, and financial improvement determine the shape of childhood development [5]. Work-life balance, time spent caring for children, and the presence of chronic illness are independent predictors of parent-child relationships [6]. Therefore, family care is also a crucial factor in health, especially in meeting nutritional needs, to improve children’s health status and growth [7].

Parental childcare is a factor associated with the nutritional status of families [8]. Since Indonesia has a 21.6% prevalence of stunting, the country’s government has directly focused on childcare and nutritional status and has produced several instructions to optimize child growth [9]. Excellent childcare must follow an overview of the nutritional status of toddlers and be an instrument for monitoring and evaluating the achievement of specific indicators and interventions issued by the Health Research and Development Agency of the Ministry of Health. The Indonesian Nutritional Status Survey [*Survey Status Gizi Indonesia* (SSGI)], which applies to provincial and city/district levels, was started in 2019 and will continue until 2024. These specific nutritional indicators include prenatal checks, routine and complete basic immunizations, monitoring of toddler growth, administration of iron supplement tablets for pregnant and young women, access to medicines by toddlers, and provision of additional food to toddlers and pregnant women. Optimal nutritional status will be obtained by children when they have easy access to nutrient-rich food, adequate healthcare services, and a healthy environment [10], especially at 1000 days of fetal life [11, 12]. Complementary breastfeeding supports healthy growth and development [13]. Poor complementary feeding has been identified as a risk factor that is directly related to nutritional problems [14]. A systematic review also showed that childcare is related to growth. There is a positive association between childcare attendance and growth, including an association with stunting [15].

**Fig. 1:**
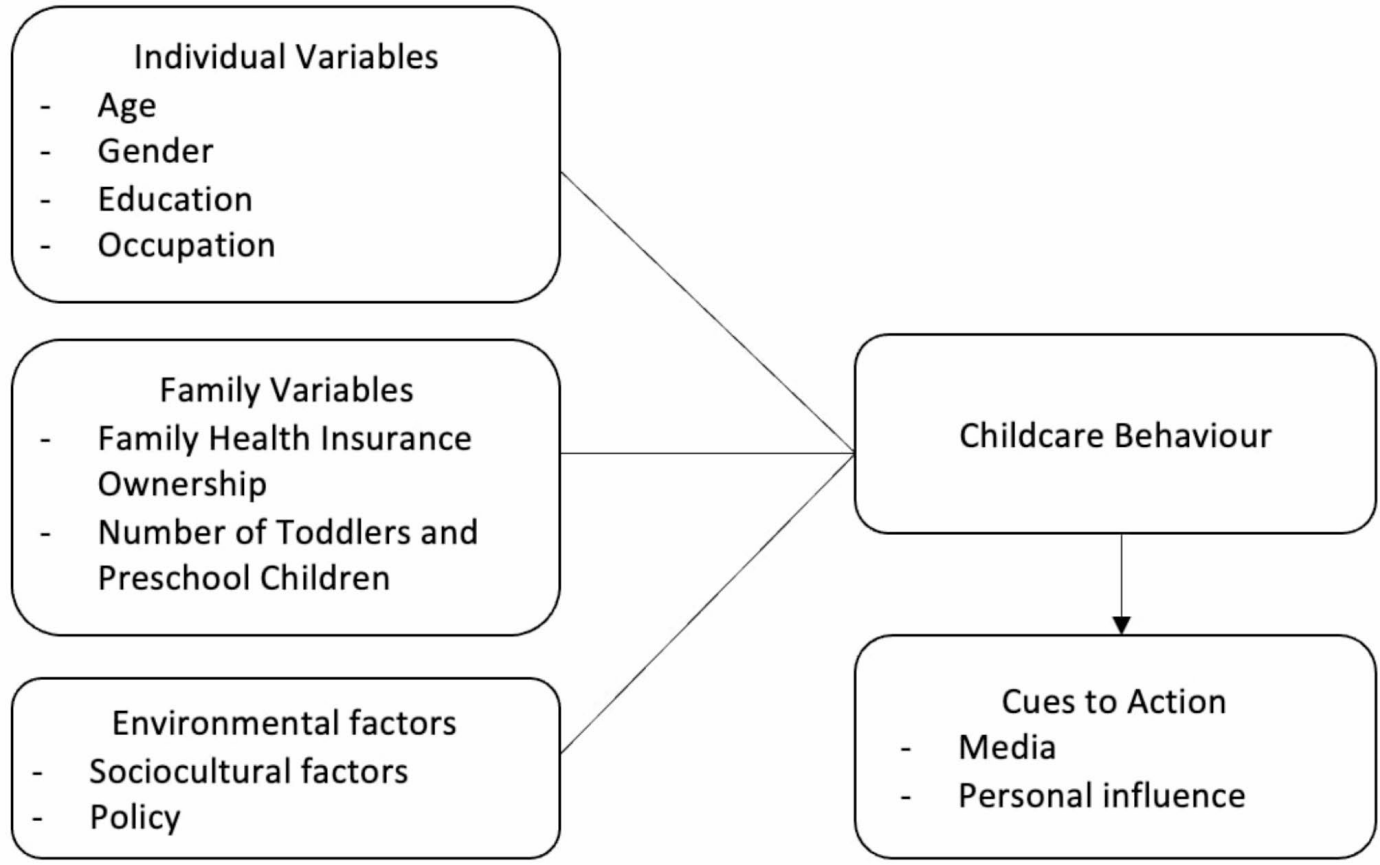
Conceptual framework

There are many risk factors believed to be the causes of nutritional problems, which occur at several levels, namely, community, family, household, and individual levels [16]. At the community level, the causes are inadequate health services, environmental cleanliness, and little clean water. Causes at the family level include economic factors, nutrition, and parenting style. Causes of individual elements included history of infectious diseases, history of weight, low birth weight, and maternal nutritional status during pregnancy [17]. Therefore, child development outcomes are related to parental knowledge, attitudes, and practices in managing family health and nutrition [18]. Another study on similar issues revealed that family income, ethnicity, or age differences may contribute to differences in childcare [19]. In particular, healthy parenting measures the extent to which parents play a role and function in carrying out their duties and responsibilities to provide good health status for their children. The description of the concept used in this study is based on the Health Belief Model, as shown in Fig. 1.

Multidisciplinary professionals conducted the SKAP survey. However, the large amount of data acquired was only used for social sciences research. Hence, specific explanations of childcare, psychology, and public health aspects have not been explored much. However, issues of stunting and malnutrition require extensive support from national-level data, such as the SKAP data survey. These data can be used to describe how childcare is influenced by various family characteristics, such as parents’ educational background, employment, insurance ownership, gender, age, number of children, and media exposure to child health. Therefore, this study aimed to analyze the relationship between family characteristics and childcare in children under six years of age based on secondary data.

## Methods

### Study design and setting

This research was conducted by the National Population and Family Planning Board for Indonesia in a cross-sectional manner, using secondary data from the Performance and Accountability Survey Program (SKAP) in 2019. The second paragraph explains the procedure for collecting primary data, and the third paragraph explains the secondary data used in this study.

SKAP is a survey and national research conducted by the National Population and Family Planning Board for Indonesia through the Center for Research and Development for Family Planning, Prosperous Families, and Population to measure the success of the family and population development programs that have been carried out. While collecting primary data, the sampling methods used were probability-proportional to size (PPS) and systematic random sampling. First, PPS sampling was used with the number of households, which resulted in household listings. PPS is a method for selecting cluster samples proportionally, considering differences in the number or size of each target. Here, “size” refers to the number of households to be included as samples. The PPS method is also used to determine both the selected cluster and its location. A cluster, in this context, is a group of adjacents adjacent census blocks, located in one area and consisting of an average of 200 households. This cluster forms part of a village or ward area with identifiable administrative boundaries. The household listings were then selected by systematic random sampling to be chosen as respondents. Households identified during data collection that meet the minimum requirement of consisting of at least one family are considered eligible. In each cluster, 35 eligible households will be interviewed. Single-person households or those that do not meet the family requirement are excluded from eligibility.

Data were collected through door-to-door interviews with the selected respondents using the SKAP questionnaire, which included questions about child growth and development. The survey was conducted in 34 provinces of Indonesia. In addition, no respondent received any intervention. Therefore, the results of the survey represent data at the Indonesian national level [20]. The data were approved by the National Population and Family Planning Board for Indonesia Ethical Review Institutions (number 454/LB.02/H4/2019).

This study uses secondary data for analysis based on the survey data obtained. The family criteria in this survey refer to Law No. 52 of 2009, concerning population and family development, the smallest unit in a society consisting of husband and wife, husband and wife with their children, fathers with their children, and mothers with their children (*Undang-Undang Republik Indonesia*, 2009). The family respondent sample comprised all families on the list of selected household members. Based on SKAP survey data, this research focuses on the two most populous provinces in Indonesia, namely West Java and Banten, with a total of 10.731 respondents’ data collected. Missing data occurred when respondents did not fill the answer column; therefore, only complete data were used for analysis. Thus, 102 respondents were excluded from the study. After being selected with research criteria, a total of 7.651 respondents in the secondary data were analyzed in this study.

### Participants

The respondents in this study were families in all provinces of Indonesia. In detail, the inclusion criteria of the respondents were couples of childbearing age families, reproductive age, or *Pasangan Usia Subur* (PUS) who were or had ever engaged in caring for children under six, with a minimum age of 14 years. There were no exclusion criteria for this study.

### Variables

Two types of variable were used in the analysis. Childcare was the dependent variable. In this study, childcare was defined as the care provided by parents. The seven indicators of SKAP were adapted from the practice of caring for the growth and development of children and toddlers, knowledge, and information related to family functioning. These indicators formed the instrument in the form of a questionnaire. The answer choices for these questions were yes and no. Indicator values of 0 to 2 yes answers indicated fairly childcare, while 3 to 7 indicated good childcare provision. Those indicators were asked to be answered based on the parents’ experience of providing caregiving to children, without considering the number of children or the child.

The independent variables in this study were family characteristics, such as age, gender, education, occupation, and media exposure to children’s health, as well as family aspects such as health insurance ownership and the number of toddlers and preschool children. Respondents of this study were only one of the parents or a respondent who represented the family parenting pattern in the household and who were available for the survey interview. In addition, media exposure is defined as the history of knowing about the national family program through health officer visits, television, posters, or other media exposures. The family health insurance ownership question asked was the BPJS National Health Insurance. Additionally, the number of toddlers under six entails the history of all respondents having children under six. Those with children more than six years old were categorized as no toddlers or children of preschool age, and they were asked a retro question to review their experience of having children under six.

### Statistical analysis

Univariate and bivariate data analyses were performed using data-processing software. Because this study has a large volume of data produced, the researcher used STATA software. This is intended so that the analysis process can be carried out optimally. Univariate analysis was carried out using the frequency distribution method, describing family characteristics and caring for children under six years in frequency (n) and proportion (%). In this analysis, the 75th percentile was used to justify the conditions of the studied variables. Bivariate analysis was performed using the chi-square test (*X*^*2*^) to assess the relationship between childcare upbringing and distinct family components. Other analyses were performed using the multivariate logistic regression method to estimate the magnitude of the probability of a particular event as an explanatory function. A multivariate regression model is a regression model with more than one correlated response variable and one or more predictor variables (OR [95% C.I]). We conducted the analysis using statistical software and then looked for a connection between variables. In this case, the possible childcare factors were determined based on family characteristics. In addition, we analyzed the data guided by the National Population and Family Planning Board’s framework of children’s growth and development, which explains the importance of monitoring children’s growth and development from the perspective of physical growth, development, and mental-emotional aspects [21].

## Results

Based on the participants’ baseline data, Table 1 shows the results of the distribution of family characteristics from a total of 7.651 households with couples of childbearing age families (PUS) samples. The highest age category, namely 2,504 samples (32.7%), was 35–49 years. This range is considered an adult and productive age category. Most of the data showed the last education category attended was primary school, comprising 3,158 samples (41.3%), and 4,208 (55%) of the sample were employed. Conditions also show that 7,415 samples (96.9%) have less than two children under six, while only 4,735 (61.9%) samples have insurance. The exposure of the sample to media that provides information about stunting is dominated by the range of 1–5 types of media, or approximately 7,138 (93.3%) of the total sample. It can be seen in Table 1.

**Table 1:** Baseline Data of participants (*n* = 7.651)

Family Characteristics	Frequency	Percentage
**Parents Age**		
< 19	805	10.5%
20–34	2.309	30.2%
35–49	2.504	32.7%
> 50	2.033	26.6%
**Parent’s Gender**		
Male	3.910	51.1%
Female	3.741	48.9%
**Parent’s Education**		
Never and not yet in school	136	1.7%
Primary School	3.158	41.3%
Junior High School	1.442	18.9%
Senior High School	2.251	29.5%
D1/D2/D3/Academy	193	2.5%
College/University	471	6.1%
**Parent’s Occupation**		
Employed	4.208	55%
Unemployed	3.443	45%
**Family Health Insurance Ownership**		
Don’t have insurance	2.916	38.1%
Have insurance	4.735	61.9%
**Parent’s Media Exposure Related to Children’s Health**		
Not exposed to media	124	1.6%
Exposure to 1–5 types of media	7.138	93.3%
Exposure to 6–10 types of media	343	4.5%
Exposure to 11–15 types of media	46	0.6%
**Number of Toddlers and Preschool Age Children (< 6 years)**		
< 2 children	7.415	96.9%
> 2 children	236	3.1%

Table 2 shows the results of the Pearson Chi-square analysis, which states a significant relationship between the variable components of family characteristics: age (ρ = 0.000), education (ρ = 0.000), health family insurance (ρ = 0.010), number of toddlers and pre-school age (ρ = 0.000) and media exposure to health information (ρ = 0.001), with childcare. With the highest category percentage that has an impact on good childcare, there were found that parents aged 20–34 years were 807 (34.9%) respondents, do not have family health insurance were 626 (21.5%) respondents, exposure to 11–15 types of media related to children’s health were 12 (26.1%) respondents and had > 2 children were 159 (67.4) respondents. However, in contrast to other family characteristic components, gender (ρ = 0.497) and occupation (*p* = 0.370) have no relationship with childcare in children. It can be seen in Table 2.

**Table 2:** The correlation between family characteristics and childcare (*n* = 7.651)

Family Characteristic	Variable	Childcare				*Chi-Square*
		Good*		Fairly*		
		Frequency (n)	Percentage (%)	Frequency (n)	Percentage (%)	
**Parent’s Age (years)**	< 19	98	12.2%	707	87.8%	0.000
	20–34	807	34.9%	1.502	65.1%	
	35–49	590	23.5%	1.914	76.5%	
	> 50	33	1.6%	2.000	98.4%	
**Parent’s Gender**	Male	769	19.7%	3.141	80.3%	0.497
	Female	759	20.3%	2.982	79.7%	
**Parent’s Education**	Not yet in school	8	5.9%	128	94.1%	0.000
	Primary School	475	15.05%	2.683	84.95%	
	Junior High School	380	26.4%	1.062	73.6%	
	Senior High School	501	22.3%	1.750	77.7%	
	D1/D2/D3/Academy	51	26.4%	142	73.6%	
	College/ University	113	24%	358	76%	
**Parent’s Occupation**	Employed	856	20.3%	3.352	79.7%	0.370
	Unemployed	672	19.5%	2.771	80.5%	
**Family Health Insurance Ownership**	Don’t have insurance	626	21.5%	2.290	78.5%	0.010
	Have insurance	902	19%	3.833	81%	
**Parent’s Media Exposure Related to Children’s Health**	Not exposed to media	8	6.4%	116	93.6%	0.001
	Exposure to 1–5 types of media	1.429	20%	5.709	80%	
	Exposure to 6–10 types of media	79	23%	264	77%	
	Exposure to 11–15 types of media	12	26.1%	34	73.9%	
**Number of Toddlers and Preschool Age Children (< 6 years)**	< 2 children	1.369	7.9%	6.046	92.1%	0.000
	> 2 children	159	67.4%	77	32.6%	

^*^ Condition of child caring for the growth and development of children is based on the 75th percentile justification

The results of the multivariate analysis showed that variables had a significant effect on childcare at a significance level of 10% (α = 0.10), except parental education. The analysis indicated that as age increased, childcare improved, with the odds of the good childcare are 3.515 times greater at the age of 20–34 years compared to the odds for those who were in the age of 14–19 years old (95% CI: 2.78–4.44, p-value = 0.000). Regarding parental educational background, the analysis showed that while parental education was not significantly associated with childcare, it still had exhibited a positive correlation. Our research finds that families without insurance have a better chance of doing good childcare compared to families who have insurance (OR: 0.853, 95% CL: 0.75–l0.96). Media exposure also plays a role, as parents who are more exposed to media tend to provide better childcare about children’s health than those with limited or no exposure to media. The optimal condition was observed among those exposed to 11–15 types of media, with an odds ratio (OR) of 2.919 (95% CI: 1.01–8.38, p-values = 0.047). Furthermore, parents with more than two toddlers and pre-school age (< 6 years old) exhibited better childcare than those with fewer than two toddlers and preschool-age, including none at all, with an odds ratio (OR) of 5.612 (95% CI: 4.20–7.48, p-value: 0.000) times the odds for those who were have less than 2 children. The results are presented in Table 3.

**Table 3:** Logistic regression of good childcare by potential Associated factors (*n* = 7.651)

Family characteristic	OR	[95% C.I]		*p*
		Lower	Upper	
**Parents Age**				
*<19				
20–34	**3.515**	2.781	4.442	0.000
35–49	2.168	1.705	2.757	0.000
> 50	0.129	0.085	0.195	0.000
**Parent’s Education**				
*Never went to school				
Primary School	1.360	0.631	2.929	0.432
Junior High School	1.611	0.744	3.486	0.225
Senior High School	1.424	0.660	3.075	0.367
D1/D2/D3/Academy	1.883	0.817	4.338	0.137
College/University	1.507	0.680	3.338	0.311
**Family Health Insurance Ownership**				
*Don’t have insurance				
Have insurance	0.853	0.753	0.966	0.013
**Parent’s Media Exposure Related to Children’s Health**				
*Not exposed to media				
Exposure to 1–5 types of media	2.468	1.149	5.298	0.020
Exposure to 6–10 types of media	2.376	1.059	5.332	0.036
Exposure to 11–15 types of media	2.919	1.016	8.383	0.047
**Number of Toddlers and Preschool Age Children (< 6 years)**				
*<2 children				
> 2 children	5.612	4.209	7.481	0.000

*Base categories

## Discussion

Factors significantly related to childcare in the present study were parents’ age, education, family health insurance ownership, media exposure related to children’s health, and the number of toddlers and preschool children. Most studies report each component of childcare separately, such as nutrition and immunization, rather than childcare as a single variable. However, parents are an important part of the adult population because they are in charge of their health of their children. Child care consists of providing children with balanced nutrition, immunizing children, providing breast milk and vitamins, treating children when sick, and teaching children healthy behaviors.

Parent education correlates with children’s nutrition by imparting literacy and skills and exposing prospective parents to health information and knowledge [22]. In this research, parental education showed a positive correlation with childcare, and media exposure to health (11–15 types of media) showed a better childcare in children. In addition, parents’ high level of health literacy was linked to healthy habits in their children, such as eating better, brushing their teeth more often, and being more active. Children under age 11, whose parents had high health literacy, consumed more vegetables, salads, and fruits than other children [23]. Dietary and physical activity patterns typically develop during childhood and persist throughout adulthood. Thus, it is crucial to develop health-promoting lifestyle patterns at an early age to promote lifelong health and to reduce the risk of lifestyle-related chronic diseases.

Health-conscious parents were more likely than non-health-conscious parents to view their child’s diet as an essential contributor to future health. Childhood diet influences the risk of developing a preference for unhealthy foods, being overweight, having poor growth and development, and developing diabetes. Parents’ health consciousness influences their perspectives and behaviors regarding their child’s nutrition and health [24].

Immunization safeguards children from infectious diseases that can be fatal. However, parental vaccination non-adherence remains high. Significant correlations were found between infant immunization practices and parental education, primary school, secondary school, and higher education, and parents who had adequate knowledge of infant immunization [25]. The factors associated with immunization adherence include vaccination-related knowledge, employment status, educational level, economic status, and family size [26]. In addition, male parents, parents who did not know anyone who had personally experienced severe side effects from any vaccine, and parents who did not believe vaccines were effective methods to protect communities from disease were significantly less likely to have vaccinated their offspring than female parents, those who knew someone who had, and those who believed that vaccines were an effective means of protecting communities from disease [27]. Incomplete immunization mothers were young, single, had secondary education, poor, and primiparous [28]. There was a significant correlation between immunization non-compliance and parental age, source of information, education, and occupation [29]. Mothers’ compliance with child immunization was correlated with their marital status, knowledge, and attitude toward immunization. Knowledge of the mother was the most accurate predictor of immunization compliance [30]. Vital to a successful child immunization program is the knowledge gained through appropriate health education from health workers regarding immunization, which will correct misconceptions and provide accurate facts and a thorough understanding of child immunization.

Exclusive breastfeeding is provided for infants for the first six months and then continuing breastfeeding for up to two years while gradually introducing other meals suitable for their age. In Indonesia, exclusive lactation is positively influenced by mothers’ level of education [31]. Indicators of exclusive breastfeeding included the household affluence index and the mother’s occupation [32]. A total of 52.3% of infants were exclusively breastfed, and the likelihood of exclusive breastfeeding significantly increased with parity, antenatal care visits, early initiation of lactation, low-income households, and living in rural areas [33]. In contrast, exclusive breastfeeding was linked to a mother’s lack of formal education, monthly income under $100, female children, absence of antenatal guidance on exclusive nursing, and lack of husband support [34]. In addition, working status, cesarean delivery, and cesarean section were associated with a decreased likelihood of exclusive lactation [33]. Interventions to promote breastfeeding in Indonesia should pay particular attention to women who are vulnerable to breastfeeding and should also be encouraged by nutrition-sensitive programs such as postpartum care and smoking cessation. Given the individual and social context, breastfeeding promotion strategies should emphasize increasing breastfeeding mothers’ knowledge and problem-solving skills, especially in urban areas where barriers to exclusive breastfeeding are more complex [35].

Children are at risk for vitamin A deficiency, along with subpopulations at risk for deficiencies in folate, thiamine, vitamin B12, niacin, riboflavin, other B vitamins, and vitamin D [36]. To combat micronutrient deficiencies, it is essential to identify those at risk and prevent and manage these risks. In addition to improved and varied diets, fortification, and other public health support measures, the public health approach includes dietary supplementation.

High parental health literacy is associated with positive health behaviors in children, such as increased consumption of fruits and vegetables, decreased consumption of sugary beverages, regular dental brushing, and increased physical activity [23]. Parents, as the most influential factors on their child’s adult health, viewed diet and physical activity in adulthood, and physical activity in infancy and adolescence as important.

The factor that did not contribute to childcare was parents’ sex. However, parental gender considerably moderated the positive parenting influence (maternal vs. paternal) [37]. Nurses and healthcare professionals play a vital role in forming partnerships with parents, addressing patient education, and assisting parents in the growth and development of children. Through the dissemination of educational materials for parents of children, the degree of knowledge could be increased to accurately identify children’s health symptoms, make healthy dietary and health behavior choices, and manage sick children. It is essential to continue to provide those involved (parents and children) with support, knowledge, and skills. There is a need for culturally and contextually pertinent educational interventions, such as traditional (herbal and religious) knowledge and evidence-based knowledge. Households must be thoroughly equipped with awareness, knowledge, and abilities. Future research must continue to elucidate the educational requirements and supportive elements for children and their parents, who are primarily responsible for their growth and development care in the home. This can guide research and clinical efforts to identify effective interventions for parents and children.

## Study limitation

This study did not evaluate the impact of a program but only captured the results (output) achieved in 2019. The availability of data obtained only from primary data collection makes secondary research limited. Some of the limitations is that not all the information related to the variables studied is available and secondary data users cannot intervene in the primary data collection process. Researchers can only analyze the data obtained based on secondary data, and the data analyzed were obtained in accordance with the research objectives. Therefore, some information, such as aspects of housing and economic status, including family income and expenditure, are not discussed in their entirety. These factors may be weaknesses of this study. However, the comprehensive and relevant nature of the SKAP survey instrument did not affect the quality of the secondary research conducted.

The results of this study suggest that the government should focus on improving education and family productivity, including family financial and social aspects, as well as massive health promotion for parents to be prepared to provide optimal childcare in the future. Therefore, these results can be used to evaluate the implementation of the National Population and Family Planning Board. In addition, it can be an input for policymakers in formulating Family Planning and Development Program policies and preparing future program implementation strategies.

## Conclusions

Child health problems occur at several levels, such as individual, family household, and community levels. As a result, the variables of parents’ education, occupation, health insurance ownership, number of toddlers, and health media exposure contribute to the quality of childcare. Meanwhile, the variables of parental gender and parents occupation did not significantly contribute to the quality of childcare. The results of the multivariate analysis showed that variables in this research had a significant effect on childcare at a significance level of 10% (α = 0.10), except parents education. Older parental age (20–34), exposure to health media (11–15 types of media), having more than two toddlers and pre-school age, and without health insurance showed better childcare. These results can be used as evaluation and input for implementing family planning and development programs in the future. Based on this study, it is recommended that the government and public health officers specifically provide more health promotion of childcare to populations with the same conditions as the related factors. It is hoped that future research will specifically look at children’s factors regarding their psychological and physical growth and development, so that they do not only focus on parental factors.

## Data Availability

No datasets were generated or analysed during the current study.
